# Development and validation of a dispersive liquid–liquid microextraction method for the determination of phthalate esters in perfumes using gas chromatography-mass spectrometry

**DOI:** 10.1039/c8ra03488e

**Published:** 2018-07-30

**Authors:** Ahmed Mostafa, Heba Shaaban

**Affiliations:** Department of Pharmaceutical Chemistry, College of Clinical Pharmacy, Imam Abdulrahman Bin Faisal University King Faisal Road, P.O. Box 1982 Eastern Province Dammam 31441 Kingdom of Saudi Arabia ammostafa@iau.edu.sa ammostaf@uwaterloo.ca +966 562623776

## Abstract

A simple, rapid, sensitive and eco-friendly method has been developed for the simultaneous determination, preconcentration and extraction of phthalate esters (dimethyl phthalate, diethyl phthalate, dibutyl phthalate, benzyl butyl phthalate and bis(2-ethylhexyl) phthalate) in perfumes using dispersive liquid–liquid microextraction (DLLME) coupled to gas chromatography-mass spectrometry (GC-MS). Various factors affecting the DLLME efficiency, including the type and volume of the extracting and dispersing solvents, salt addition and vortex extraction time were optimized. Under optimum conditions, the proposed method provided low detection limits (0.003–0.070 ng mL^−1^), high extraction recovery (85.6–95.8%), wide linearity range (1.0–1000 ng mL^−1^) with good regression coefficients (>0.9984) and good precision (RSD% < 4.2%). The optimized method was successfully utilized for the analysis of different branded perfume samples and satisfactory results were obtained.

## Abstract 2

DLLME coupled to GC-MS method has been developed, optimized and validated for the simultaneous determination of phthalates in perfumes.

## Graphical abstract

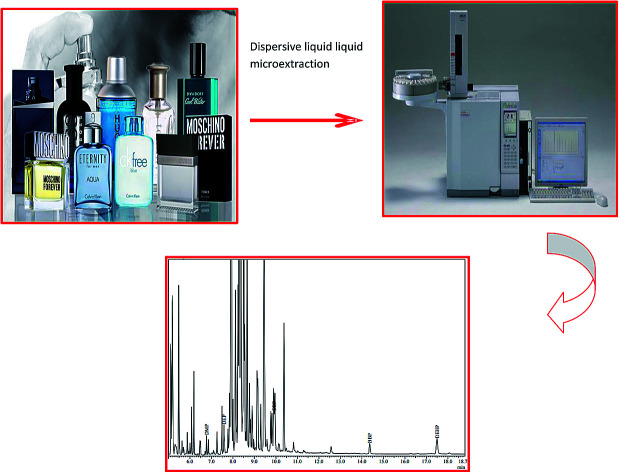

## 1. Introduction

Endocrine disrupting compounds are chemicals that can disrupt the functions of the endocrine system.^1^ These chemicals include phthalates, parabens, alkylphenols, polychlorinated biphenyls, heavy metals, *etc.* Humans are exposed to endocrine disrupting compounds from many sources such as food, water, and personal care products.^2^ Recently, these compounds have received special attention due to their widespread use in human life with possible estrogenic activity^3–5^ and negative impacts on the environment.^6–8^

Phthalate esters (PEs) are widely used in various products. For example, high molecular weight PEs are used in the manufacturing of polyvinyl chloride (PVC) plastics as additives and plasticizers. On the other hand, PEs with low molecular weight are used as solvents or adhesives in the production of wax and ink.^9^ They are commonly used as solvents and odorless diluents in cosmetics and they are also known to be used in fragrances as solvents and fixatives.^10^

Topical exposure to PEs in cosmetic products may contribute to the observed urinary levels of their metabolites in humans. A survey conducted in 2004 reported high levels of the mono-esters of certain PEs in the urine of the U.S. population.^11^ In particular, many toxicological studies indicated an association between certain PEs such as dibutyl phthalate, benzyl butyl phthalate, and bis(2-ethylhexyl) phthalate and disruption of the reproductive tract development in human male infants.^12^ Because of the concerns over health effects of PEs, different policies and regulations have been developed to restrict their use. For example, the European Union prohibited the use of dibutyl phthalate, benzyl butyl phthalate and bis(2-ethylhexyl) phthalate in cosmetic products.^13^

Several analytical methods have been developed for the determination of PEs in cosmetic products.^9,14–27^ The most commonly used technique for the determination of PEs in perfumes is gas chromatography – mass spectrometry (GC-MS) *e.g.*^9,18,19,28–31^

Several studies have reported the presence PEs in perfumes in wide concentration ranges from sub ppm to high percentage.^31,32^ Therefore a preconcentration step is usually required in order to determine PEs at sub ppm levels.^33^ Different sample preparation techniques such as direct dilution,^19^ vortex extraction,^28^ ultrasonic extraction treatment using organic solvents^18,22,29^ or solid phase microextraction^31^ have been used for extracting PEs.

Recently, dispersive liquid–liquid microextraction (DLLME) sample preparation technique has attracted many researchers in analytical chemistry community. This technique was first proposed by Rezaee *et al.* in 2006.^34^ It is based on a ternary-component solvent system composed of the extraction solvent, dispersing solvent and aqueous sample. Using additional dispersing solvent allows the formation of a cloudy solution and consequently results in a large area of contact between the extraction solvent and the aqueous sample solution leading to a rapid transfer of the analyte from aqueous solution to the extraction solvent and completion of the sample preparation in a short time.^34^ Because of the great advantages of this technique such as high recovery, ease of operation, low cost and rapidity, DLLME is preferred by many researchers.^35,36^ Despite of the widespread applications of DLLME, to the best of our knowledge, no application using DLLME coupled to GC-MS has been reported for determination of PEs in perfumes.

The aim of this work is to develop and optimize a DLLME method coupled to GC-MS for the determination of PEs in perfumes. The developed method was validated and then applied to twelve different perfume samples. The developed method requires only few seconds for sample extraction and consumes few microliters of organic solvents. Overall, the developed method is rapid, simple, sensitive and can be used as a green alternative for the analysis of PEs in perfumes and other matrices.

## 2. Materials and methods

### 2.1. Reagents and standards

All PEs standards including dimethyl phthalate (DMP), diethyl phthalate (DEP), dibutyl phthalate (DBP), benzyl butyl phthalate (BBP) and bis(2-ethylhexyl) phthalate (DEHP) were purchased from Sigma Aldrich, Germany. The purities of all PEs were >98%. A 1000 μg mL^−1^ stock standard solution of each standard was prepared in methanol and stored in glass vials at −20 °C. The standard working solution mixtures (0.001–1.00 μg mL^−1^) were prepared daily by diluting appropriate aliquots of the stock standard solutions to obtain the required concentrations and stored at 4 °C. HPLC grade methanol, acetonitrile and acetone were purchased from Sigma-Aldrich, Germany. Chloroform (CHCl_3_), dichloromethane (CH_2_Cl_2_), chlorobenzene (C_6_H_5_Cl), carbon tetrachloride (CCl_4_) and sodium chloride (NaCl) were from Merck (Darmstadt, Germany). Deionized water (18.2 mΩ cm) was prepared by Pure Lab Ultra water system (ELGA, High Wycombe, UK) and used in all procedures. Glass tight microsyringes (Hamilton, Nevada, USA) were used for measuring the extracted sediment volume and for the DLLME procedure. All glassware used were rinsed with deionized water then acetone before drying at 300 °C for at least 5 hours. Moreover blank runs were done for each set of samples.

### 2.2. Instrumentation

The analysis was performed using a Shimadzu (Japan) 2010 plus gas chromatograph equipped with a split/splitless injector and a QP2010 Ultra mass spectrometric detector. The MS was operated at the electron ionization (EI) mode (70 eV). The injection was operated in the splitless mode with an injector temperature of 270 °C. The analytes were separated on 30 m × 0.25 mm i.d. × 1.00 μm film thickness Rxi-5MS capillary column (Restek Corporation, Bellefonte, PA, USA) using helium (99.999%) as carrier gas with a flow rate of 1 mL min^−1^. The initial oven temperature was held at 90 °C for 0.5 min and subsequently ramped to 220 °C at 30 °C min^−1^, then increased to 280 °C at 15 °C min^−1^ and held for 10 min. The MS ion source temperature and transfer line were kept at 250 °C and 280 °C, respectively. A solvent delay time of 5 min was used. In order to obtain maximum sensitivity and selectivity, the MS was operated in selective ion monitoring (SIM) mode. Table 1 shows the SIM parameters and retention times of each analyte. Confirmation of the PEs was achieved based on the retention times and the relative abundances of the monitored ions (Table 1). Shimadzu GCMS Solution® version 2.71 was used for data acquiring, processing and GC-MS control.

**Table 1:** Molecular weight (MW), retention times and selected ions of the PEs studied by GC-MS

Compound	MW	Retention time (min)	Selected ions (*m*/*z*)	
			Quantitative ion (*m*/*z*)	Qualitative ions (*m*/*z*)
DMP	194	6.75	163	77, 194
DEP	222	7.56	149	105, 177
DBP	278	9.90	149	104, 223
BBP	312	14.31	149	91, 206
DEHP	390	17.43	149	113, 167

### 2.3. Perfume samples preparation

Twelve different perfume samples were purchased randomly from local market (Dammam, Saudi Arabia). All samples were clear liquids therefore; no pretreatment was required apart from dilution (1 : 5) with deionized water to decrease the matrix effect on DLLME. After the first round of analysis and due to the huge range of concentration differences of PEs (*i.e.*, especially for DEP), dilutions of 1 : 10 and 1 : 50 with deionized water were also injected in some cases and reanalyzed to be within the linear range of the calibration curve.

### 2.4. DLLME procedure

A 5.0 mL of the sample solution was transferred into a 15 mL screw cap glass centrifuge tube with conical bottom. 0.5 mL of acetone as the disperser solvent, containing 20 μL of C_6_H_5_Cl as the extraction solvent was injected rapidly into the sample solution. The dispersion was then mixed using a vortex mixer for 30 seconds, and a cloudy emulsion of water/acetone/C_6_H_5_Cl was formed. Phase separation was then achieved by centrifugation at 3500 rpm for 3 min. The sedimented organic phase (9.0 ± 0.5 μL) was collected with a 10 μL syringe. Finally 1 μL was injected directly into the GC-MS. All samples were quantified in triplicate.

## 3. Results and discussion

One of the most challenging points in perfumes analysis is the wide concentration range of the target analytes (between the sub ppm and high percentage).^31,32^ Due to the high concentration of some of the target analytes (especially DEP), all real samples were diluted by a factor of 5 to 50 with deionized water to achieve the proper quantitation of all analytes. Moreover, a high dilution is usually required when analyzing perfume samples using GC-MS to avoid the introduction of high water content into the GC column.^32^ Therefore, in this work, DLLME method was developed to preconcentrate and determine PEs at sub ppm levels.

In order to optimize the extraction method, different parameters should be taken into consideration, including the type and volume of extraction and dispersing solvents, the ionic strength and the vortex extraction time. All these experimental parameters were investigated to achieve the optimal conditions. All optimization experiments were performed in triplicate using 5.0 mL deionized water spiked with 0.02 μg mL^−1^ PEs. Extraction efficiency was evaluated based on the extraction recovery (ER %) and/or enrichment factor (EF). EF was calculated as the ratio of the analyte concentration in the organic sediment to its initial concentration in the aqueous phase. The concentration in the organic sediment was calculated using the calibration curve obtained from the direct injection of each PEs in chlorobenzene in the concentration range of 3–50 μg mL^−1^.

### 3.1. Selection of the extraction solvent

**Fig. 1:**
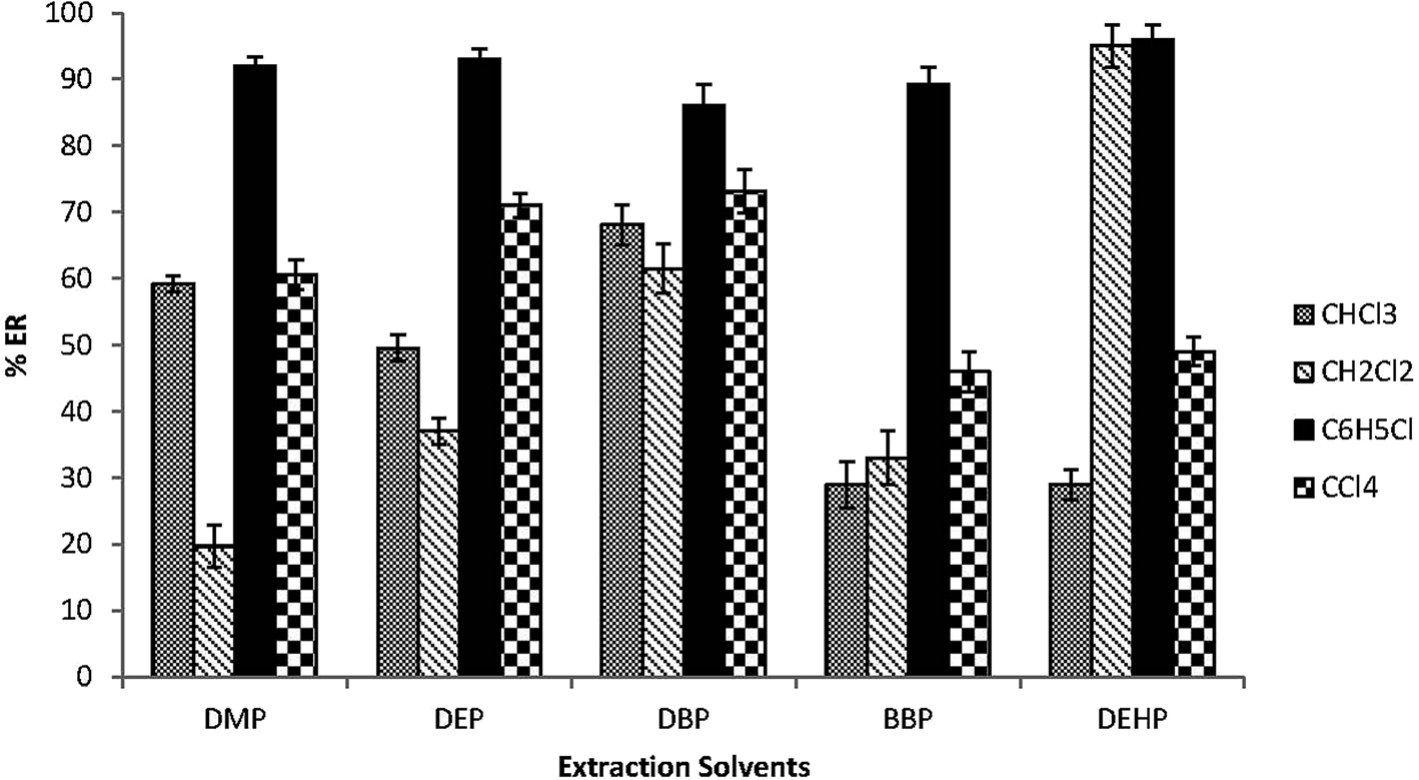
Effect of the extraction solvents on the extraction recovery (% ER) of the studied PEs. Conditions: 5.0 mL water sample volume; 0.5 mL acetone (disperser solvent); 30 μL extraction solvent volumes (chloroform “CHCl_3_”, dichloromethane “CH_2_Cl_2_”, chlorobenzene “C_6_H_5_Cl” and carbon tetrachloride “CCl_4_”); 0.02 μg mL^−1^ of each PEs.

The type of the extraction solvent has a critical role in the optimization of the extraction method to achieve good recoveries and high selectivity. The extraction solvent should have higher density than water, low solubility in water, high extraction efficiency for the target analytes and good chromatographic behavior.^37^ CHCl_3_, CH_2_Cl_2_, C_6_H_5_Cl and CCl_4_ were investigated. 30 μL of each extraction solvent and 0.5 mL acetone were rapidly injected into 5.0 mL spiked water samples in a 15 mL glass conical centrifuge tube. Fig. 1 shows that C_6_H_5_Cl has the highest extraction efficiency for the target analytes among all tested solvents. Therefore C_6_H_5_Cl was selected as the extraction solvent.

### 3.2. Selection of the disperser solvent

The disperser solvent should be miscible with the organic phase (extraction solvent) and the aqueous phase (sample solution) in order to provide extensive surface contacts between the two phases, thus enhancing the mass transfer of target analytes and improving the extraction efficiency. Based on that methanol, acetone and acetonitrile were selected for this purpose. 0.5 mL of each solvent containing 20 μL C_6_H_5_Cl were applied into 5.0 mL of spiked water samples. The performance of the three disperser solvents was very close. Therefore acetone was selected because of its lower toxicity and price.

### 3.3. Effect of extraction solvent volume

**Fig. 2:**
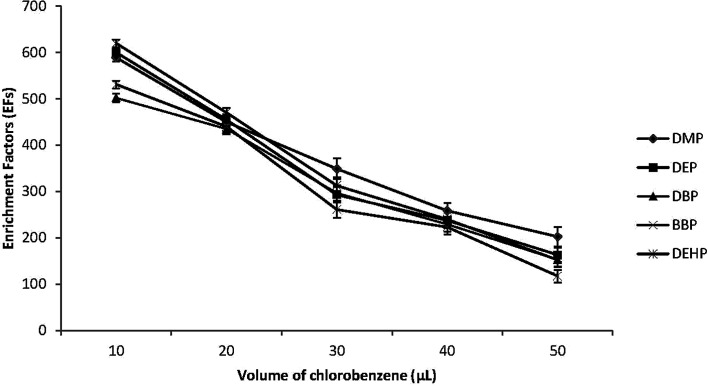
Effect of extraction solvent (chlorobenzene) volume on the enrichment factors (EFs) of the studied PEs. Conditions: 5.0 mL water sample volume; 0.5 mL disperser solvent (acetone); different chlorobenzene volumes (10, 20, 30, 40 and 50 μL); 0.02 μg mL^−1^ of each PEs.

The extraction solvent volume is another important factor that affects the efficiency of the DLLME. Therefore different volumes of C_6_H_5_Cl between 10.0 and 50.0 μL were dissolved in 0.5 mL of acetone and then applied to the DLLME procedure. Fig. 2 shows that the EFs of the target PEs decreased with the increase of the volume of the extraction solvent. By increasing the volume of C_6_H_5_Cl, the volume of the sediment phase was increased accordingly, and EFs were reduced due to dilution effect. However, when 10.0 μL volume of C_6_H_5_Cl was used, the sediment phase was hard to be removed by microsyringe and there was a drastic reduction in the reproducibility and extraction recoveries. Therefor 20.0 μL of C_6_H_5_Cl was used in subsequent experiments.

### 3.4. Effect of disperser solvent volume

**Fig. 3:**
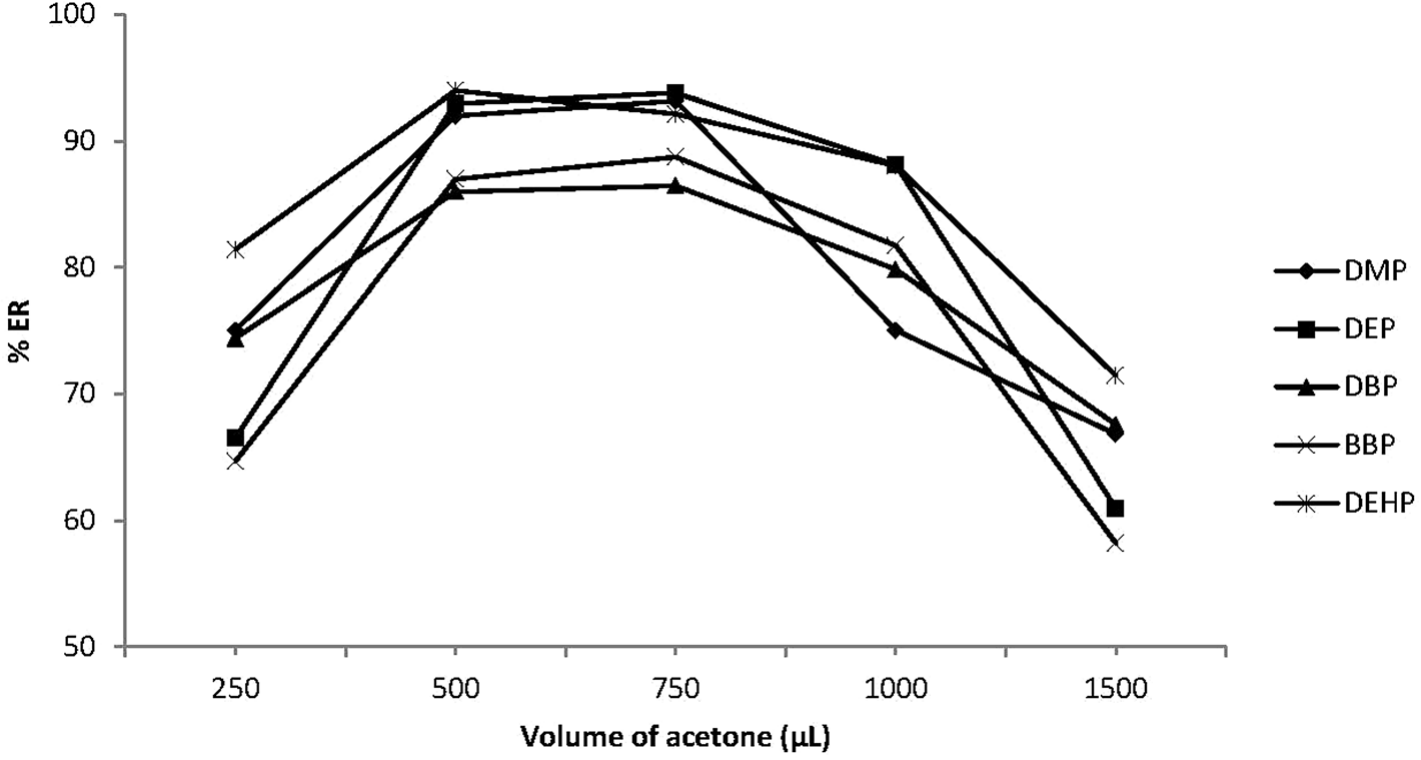
Effect of disperser solvent (acetone) volume on the extraction recovery (% ER) of the studied PEs. Conditions: 5.0 mL water sample volume; 20 μL extraction solvent (chlorobenzene); different volumes of acetone (250, 500, 750, 1000 and 1500 μL); 0.02 μg mL^−1^ of each PEs.

The volume of the disperser solvent is also an important parameter to achieve efficient DLLME. This volume should be optimized to be as little as possible to reduce the toxic effect on the environment. Meanwhile it should not be too small to enable the formation of an emulsion (water/acetone/C_6_H_5_Cl) to achieve the required degree of dispersion of C_6_H_5_Cl in the sample phase and enhance the extraction efficiency. Various volumes of acetone (0.25–1.5 mL) were applied to 5.0 mL sample solution. Fig. 3 shows that ERs (%) increased with increasing acetone volume up to 0.5 mL and then decreased with further increase in acetone volume. Small acetone volumes less than 0.5 mL were not able to enhance emulsion formation, thus the extraction efficiency was decreased. On the other hand, higher acetone volumes enhanced the PEs solubility in water, which reduced the distribution coefficients of PEs into C_6_H_5_Cl and decreased the extraction efficiency. Therefore 0.5 mL acetone was used as the optimum volume.

### 3.5. Effect of the ionic strength

Different concentrations of sodium chloride (0–15%, w/v) were investigated to study the effect of salt addition on the DLLME efficiency while keeping other experimental conditions constant. There was no significant change in ERs (%) for all analytes with the addition of sodium chloride (data not shown). The increase of ionic strength may reduce PEs solubility in water, thus enhancing their partitioning into C_6_H_5_Cl. On the other hand, this may also diminish C_6_H_5_Cl solubility in aqueous phase, thus increasing the sediment volume and decreasing the enrichment factor. Furthermore, the viscosity of the aqueous phase also increased with the increase of salt concentration, and reduced the mass transfer efficiency between the two phases.^38^ Therefore no salt was added in the developed method.

### 3.6. Effect of the vortex time

**Fig. 4:**
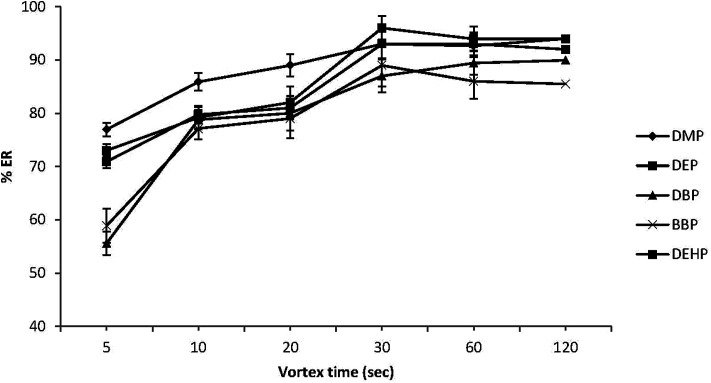
Effect of vortex extraction time on the extraction recovery (% ER) of the studied PEs. Conditions: 5.0 mL water sample volume; 20 μL extraction solvent (chlorobenzene); 500 μL disperser solvent (acetone); 0.02 μg mL^−1^ of each PEs.

Some studies have reported that vortex agitation may increase the dispersion of the extraction solvent into the aqueous solution, thus enhancing the extraction efficiency.^39–41^ Therefore, in this study different vortex times (5, 10, 20, 30, 60 and 120 s) were investigated at fixed rotational speed (2800 rpm). As can be seen in Fig. 4, the ERs (%) were increased with extension of vortex time from 5 to 30 s, but remained stable after 30 s indicating that equilibrium for all target PEs was achieved after only 30 s. In line with that, 30 s was selected as the optimum vortex time, enabling extraction under equilibrium conditions and enhancing sensitivity.

### 3.7. Validation of the method

**Fig. 5:**
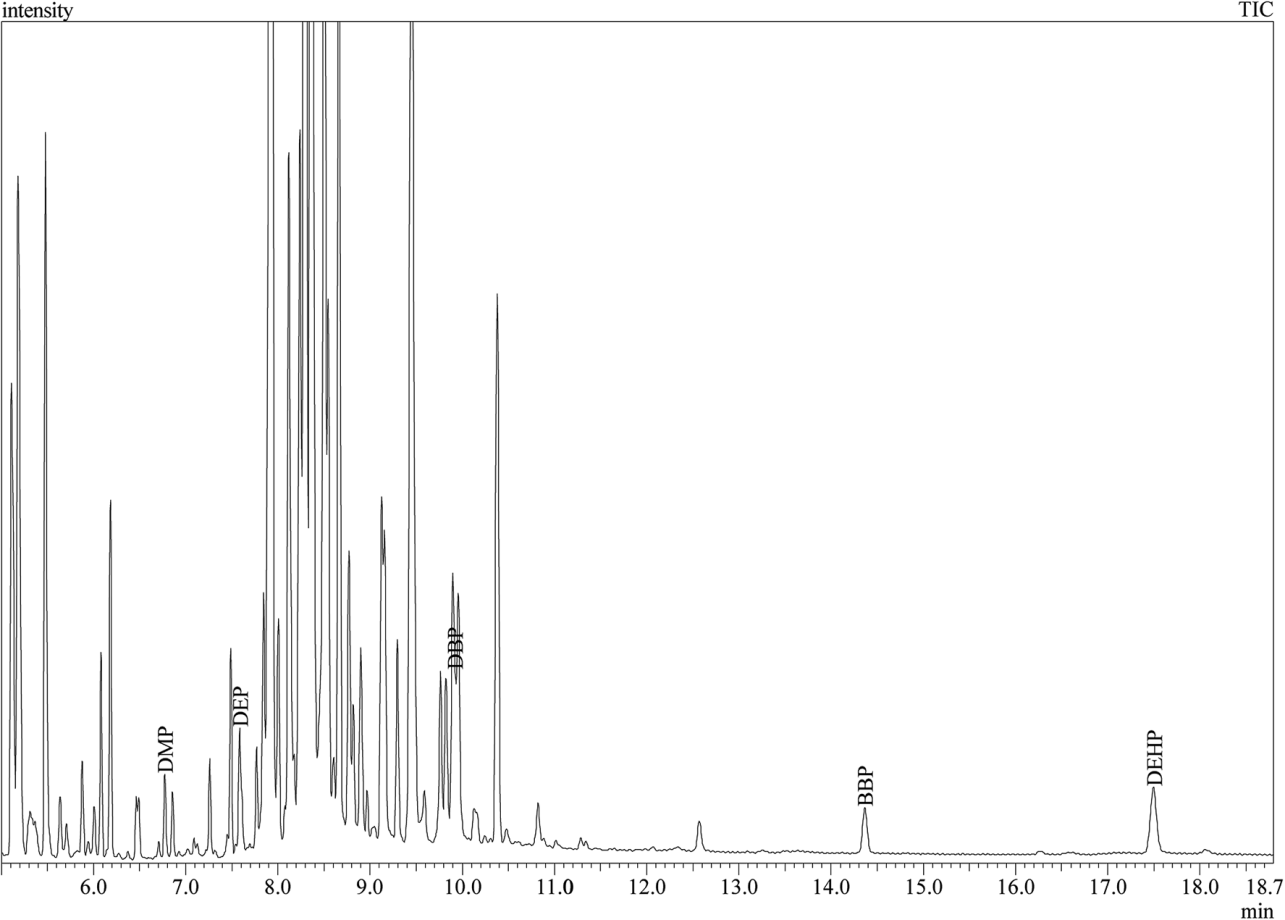
Total ion chromatogram (TIC) of the GC-MS separation of the studied PEs in perfume sample (S11 spiked with DMP and BBP).

Under the optimized conditions, the method developed was validated according to different parameters. Linearity, limit of detection (LODs), limit of quantification (LOQs), precision, accuracy, EFs and ERs were calculated and summarized in Table 2. Linearity was studied by extracting a series of standard solutions containing various analyte concentrations and the respective calibration curve was developed. Good linearity with regression coefficients (*r*^2^) higher than 0.9984 was observed for all analytes in the range of 0.001–1.0 μg mL^−1^. LODs and LOQs, which were calculated based on signal-to-noise (S/N) ratio of 3 and 10, were in the range of 3.0 × 10^−6^ to 7.0 × 10^−5^ μg mL^−1^ and 1.3 × 10^−5^ to 2.0 × 10^−4^ μg mL^−1^ for different PEs, respectively. The intra-day and inter-day precision of the method were investigated by calculating the RSD% of three replicate extractions (Table 2). As can be seen RSD% values were in the acceptable range of 1.36–4.12%. Furthermore, high EFs in the range 476–532, and ERs (%) in the range of 85.6–95.8% were achieved. The broad linear dynamic range combined with low LOQs and LODs suggest the benefits of the proposed DLLME-GC-MS method in PEs analysis in perfumes. Fig. 5 shows the total ion chromatogram (TIC) of the GC-MS separation of the studied PEs in spiked perfume sample. Good separation of the studied analytes was achieved.

**Table 2:** Quantitative parameters of the DLLME – GC-MS method for the determination of the selected PEs

Compound	Linearity range (μg mL^−1^)	*r* ^2^	LOD^a^ (μg mL^−1^)	LOQ^b^ (μg mL^−1^)	RSD^c^ %		EF^d^	Recovery^e^ (%)
					Intra-day	Inter-day		
DMP	0.0010–1.0	0.9998	5.0 × 10^−6^	2.0 × 10^−5^	1.36	2.75	510	91.8
DEP	0.0010–1.0	0.9998	6.0 × 10^−6^	2.2 × 10^−5^	1.77	2.39	513	92.3
DBP	0.0010–1.0	0.9996	3.0 × 10^−6^	1.3 × 10^−5^	3.66	3.46	476	85.6
BBP	0.0015–1.0	0.9985	7.0 × 10^−5^	2.0 × 10^−4^	3.01	4.12	487	87.6
DEHP	0.0010–1.0	0.9987	4.0 × 10^−6^	1.5 × 10^−5^	2.25	3.33	532	95.8

a Limit of detection (S/N = 3).

b Limit of quantification (S/N = 10).

c Relative standard deviation for (*n* = 3) at concentration of 0.02 μg mL^−1^ of each PEs.

d Enrichment factor.

e For water sample spiked with 0.02 μg mL^−1^ PEs.

### 3.8. Comparison of the developed DLLME method with other presented methods

Table 3 presents a comparison between the proposed method and previous published methods for the determination of PEs in different perfume and/or cosmetics. The main advantage of the proposed method is the low consumption of organic solvents compared to other liquid–liquid extraction methods. In addition the proposed method provided lower LODs and wider linear dynamic ranges. Moreover, the extracting time was very short and equilibrium was achieved very rapidly. It can be concluded that DLLME-GC-MS is a rapid, sensitive, eco-friendly and precise method that can be used for the preconcentration and determination of PEs in different perfume samples.

**Table 3:** Comparison of the proposed DLLME – GC-MS method with other methods for the determination of PEs in perfumes and/or cosmetics

Method	Matrix	Extractiontime	Extraction Solvent volume	Detection method	LODs (ng mL^−1^)	Linearity (ng mL^−1^)	Ref.
UAE-DLLME^a^	Cleaning and PCPs^b^	20 s	150 μL CCl_4_ + 3 mL acetonitrile	LC-MS-MS	0.04–0.45	0.4–50	24
UAE-DLLME	Cleaning and PCPs	20 s	150 μL CCl_4_ + 3 mL acetonitrile	LC-DAD	0.13–1.0	2.0–50	24
DLLME-SFO	Waters and cosmetics	10 min	20 μL 1-dodecanol + 30 μL acetone	LC-DAD	20.0–170.0	500.0–50 000	25
USAEME-SFO^c^	Waters and cosmetics	12 min	30 μL 1-undecanol	LC-DAD	0.005–0.01	0.05–1000	26
USESSM^d^	Waters and cosmetics	1 min	4 mL THF containing 30 mg decanoic acid	LC-UV	0.1–0.7	0.5–100	27
SPME^e^	Perfumes	23 min	—	GC-MS	0.513–0.770	0.75–24	31
DLLME	Perfumes	30 s	20 μL C_6_H_5_Cl + 0.5 mL acetone	GC-MS	0.003–0.070	1.0–1000	Represented work

a Ultrasonic-assisted extraction.

b Personal-care products.

c Ultrasound-assisted emulsification microextraction with solidification of floating organic droplet.

d Ultrasonically enhanced supramolecular solvent microextraction.

e Solid phase microextraction.

### 3.9. Perfume samples analysis

**Fig. 6:**
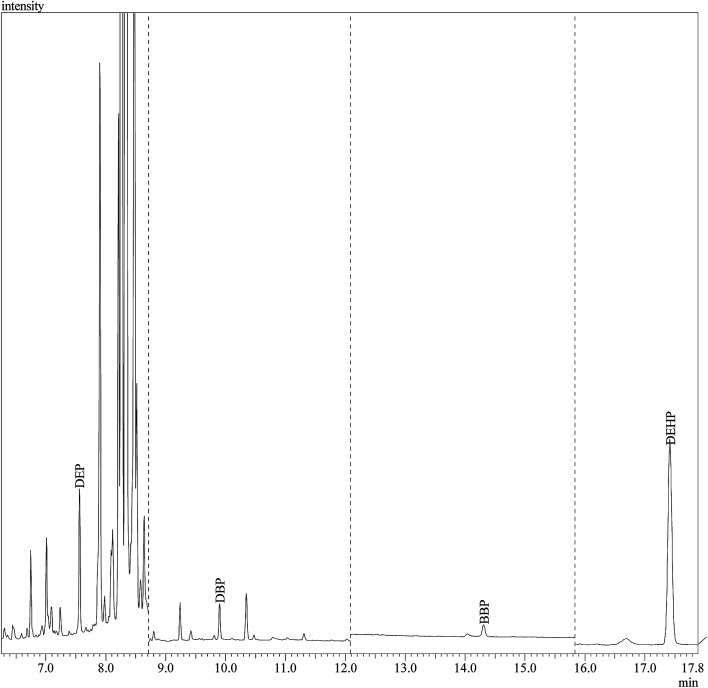
GC-MS separation of the studied PEs in sample “S11” under SIM conditions shown in Table 1.

The performance of the developed method was evaluated through its application for the determination of the studied PEs in 12 different branded perfume samples from the local market. To assess the matrix effects, the perfume samples were spiked at three different concentration levels (0.002 μg mL^−1^, 0.05 μg mL^−1^ and 0.5 μg mL^−1^) of PEs standards except for DEP and DEHP. The spiking levels of DEP were 0.002 μg mL^−1^, 0.05 μg mL^−1^ and 0.3 μg mL^−1^; and for DEHP the spiking levels were 0.002 μg mL^−1^, 0.01 μg mL^−1^ and 0.07 μg mL^−1^. Satisfactory recoveries were obtained ranging from 84.6–109.7% (Table 4) which demonstrated the applicability of the developed DLLME-GC-MS method for the determination of PEs in perfume samples. Fig. 6 shows the chromatographic separation of PEs in sample “S11”.

**Table 4:** Extraction recoveries of PEs in spiked perfume samples

Sample	Spiked amount (μg mL^−1^)	S8		S9		S11	
		Recovery (%)	RSD^a^ (%)	Recovery (%)	RSD^a^ (%)	Recovery (%)	RSD^a^ (%)
DMP	0.002	87.4	2.45	98.8	1.34	86.2	1.45
	0.05	89.4	3.00	102.9	1.91	84.6	0.70
	0.5	86.9	1.93	101.7	1.22	87.3	1.06
DEP	0.002	103.5	1.78	93.5	1.24	91.5	1.73
	0.05	101.8	2.54	94.3	1.51	92.3	1.28
	0.3	101.5	2.04	94.7	1.56	89.6	1.89
DBP	0.002	87.8	1.96	84.7	2.15	104.8	2.42
	0.05	86.5	1.08	86.5	2.07	103.2	1.99
	0.5	89.9	2.02	88.6	1.78	99.7	1.73
BBP	0.002	93.4	1.87	93.8	1.86	92.5	1.86
	0.05	89.9	2.83	91.3	2.02	92.3	0.64
	0.5	95.3	1.45	96.7	1.46	91.9	2.42
DEHP	0.002	93.2	2.10	104.3	1.98	93.5	1.74
	0.01	94.6	3.09	109.7	2.78	95.2	2.92
	0.07	94.7	2.78	103.1	1.49	97.2	1.56

a Relative standard deviation (*n* = 3).

Table 5 shows the results obtained from the analysis of the commercial perfume samples. All five PEs were found in most of the analyzed samples, with detection frequency rates of 12 out of 12 samples for DEP and DEHP, 11/12 for BBP, 10/12 for DBP and 6/12 for DMP. DEP was the most abundant analyte with concentration ranging from 0.22 to 4213 μg mL^−1^. This result is in good agreement with previous studies reporting high concentrations of DEP in perfumes.^19,31,42^ This can be attributed mainly because DEP is used as a solvent and fragrance stabilizer. Although DEHP and DBP are banned in Europe,^13^ they were found in considerable concentrations in most samples (21.68–228.54 μg mL^−1^ and ND – 25.91 μg mL^−1^, for DEHP and DBP, respectively). DMP was found in low concentrations (less than 3.55 μg mL^−1^) except for sample “S12”, where the concentration was high (16.24 μg mL^−1^). BBP was found in low concentrations ranging from ND to 3.05 μg mL^−1^.

**Table 5:** Contents of PEs (μg mL^−1^) in commercial perfumes

Sample	DMP		DEP		DBP		BBP		DEHP	
	Found (μg mL^−1^)	RSD^a^ (%)	Found (μg mL^−1^)	RSD (%)	Found (μg mL^−1^)	RSD (%)	Found (μg mL^−1^)	RSD (%)	Found (μg mL^−1^)	RSD (%)
S1	3.46	2.23	4015	0.83	6.43	2.35	0.38	2.56	34.93	1.92
S2	ND^b^	2.74	2526	1.34	ND	2.37	3.05	2.26	158.46	1.69
S3	3.54	1.89	4213	1.58	8.24	0.86	2.88	1.59	109.30	0.60
S4	ND	2.25	1191	1.99	25.91	1.87	2.77	2.92	228.54	2.06
S5	1.92	2.09	372	1.02	ND	0.90	1.18	1.73	73.77	2.53
S6	ND	0.62	3569	2.01	4.49	2.17	1.50	0.83	101.70	2.83
S7	1.64	1.99	4156	2.90	17.59	2.13	0.86	2.40	41.81	1.05
S8	0.61	2.11	30.12	2.66	2.18	3.09	0.06	0.99	21.68	0.76
S9	ND	2.84	0.22	0.75	ND	2.11	ND	2.13	46.05	3.00
S10	ND	2.41	25.66	1.36	2.97	2.10	0.50	0.86	50.95	2.73
S11	ND	1.67	8.58	1.36	2.39	1.75	0.01	0.64	28.90	2.57
S12	16.24	1.22	3752	2.86	2.18	1.38	0.12	1.50	27.44	2.08

a Relative standard deviation (*n* = 3).

b Not detected.

The presence of banned PEs contamination in high concentration is alarming and more attention should be focused on the control of the content of these substances in perfumes marketed in Saudi Arabia. However, it worth mentioning that these high concentration PEs may come from different sources such as packing of raw materials or the plastic spray tube inside the perfume bottle and not necessarily added intentionally during the manufacturing process.^9,19^

## 4. Conclusions

In the presented study a new DLLME method was developed for the preconcentration of PEs followed by their determination using GC-MS. The validation of the method was satisfactory and showed good linearity with a wide dynamic range, low LODs and LOQs, adequate accuracy and precision. This method provides a valuable fast, sensitive and eco-friendly option for the determination of PEs in different branded perfume samples.

## Conflicts of interest

The authors declare that they have no conflict of interest.

## Supplementary Material
